# Anthropometric Measures, Blood Pressure and Major Laboratory Examination Results in the Health Check-up Examination among the JPHC Study Participants at Baseline Survey

**DOI:** 10.2188/jea.11.6sup_87

**Published:** 2007-11-30

**Authors:** Shunroku Baba, Toshifumi Mannami, Masamitsu Konishi, Satoshi Sasaki

**Affiliations:** 1 Department of Preventive Cardiology, National Cardiovascular Center.; 2 Department of Public Health, Ehime University School of Medicine.; 3 Epidemiology and Biostatistics Division, National Cancer Center Research Institute East.

**Keywords:** health check-up, health center, JPHC study

## Abstract

Health check-up data were compared in all 11 populations included in the cohort. The collected sample size was 23,313 in Cohort I and 24,654 in Cohort II. Height was greater in two urban populations, and body mass index (BMI) was largest in the two populations in Okinawa prefecture. Blood pressure was higher in the populations in northeast part of Japan and Okinawa prefecture, and lower in Suita. Serum total cholesterol level was higher in Okinawa and two urban populations, and lower in the populations in northeast part of Japan, and in Arikawa and Saku.

## INTRODUCTION

Along with the questionnaires which was administered to all the target population, data was collected for health check ups carried annually by the local municipalities under the Health Services for the Elderly Act, which are inexpensive or free for all people in any area of Japan who hold a resident card, aged 40 years old and over, and who are not offered periodical health examinations at their place of work. In this paper, we tried to compare the results of the health check ups to see the difference in the basic health status in each population, though the rates of attendance to the examination were relatively low.

## METHODS

The examinations were primarily done in 1990 for Cohort I and in 1993 for Cohort II. But owing to the different conditions, data was collected differently in some populations; in Ishikawa the data was collected in the years 1990 to 1992, in Katsushika in 1990 to 1992, in Suita sub-cohort 1 & 2 in 1993 to 1994 and in Kasama in 1993 and 1994.

As for the data, the health check up data made by the municipality governments under the Health Services for the Elderly Act was collected and linked to the cohort subjects. Data from other sources were added when available. The items required by the law were blood pressure measurement, serum total cholesterol, aspartate aminotransferase, alanine aminotransferase, blood glucose, blood hemoglobin concentration, red blood cell counts, hematocrit, urinary glucose, urinary protein and urinary occult blood in 1990 when data for Cohort I was mainly collected and in addition to these, serum triglyceride, serum *γ*-glutamyl transferase and serum high density lipoprotein (HDL) cholesterol in 1993 when main data for Cohort II were collected. The proportions of the examined subjects for each item are listed in Table 1.

**Table 1.** Health check-up attendance rate per 100 among the selected sample and the examined rate per 100 of each item among the attendants in each population.

	Ninohe	Yokote	Saku	Ishikawa	Katsushika	Kasama	Kashiwazaki	Tosayamada	Arikawa	Miyako	Suita 1	Suita 2
Attendance rate(%)	40.9	32.7	43.5	16.8	–	26.2	24.5	30.7	31.7	39.1	25.8	41.3

Items												
Height	99.9	99.7	99.8	100.0	98.9	99.6	99.9	99.9	97.6	99.9	99.9	100.0
Weight	99.9	100.0	99.9	100.0	98.5	100.0	99.9	100.0	97.7	99.9	99.9	100.0
Systolic Blood Pressure	100.0	100.0	99.8	100.0	98.8	100.0	100.0	99.9	98.6	99.9	99.9	100.0
Diastolic Blood Pressure	100.0	100.0	99.8	100.0	98.8	100.0	100.0	99.9	98.6	99.9	99.9	100.0
Serum Chemicals												
Total Cholesterol	99.9	99.8	99.9	100.0	99.4	99.6	100.0	99.7	99.8	99.7	98.4	100.0
Triglyceride	21.3	19.6	96.0	15.6	45.2	99.6	100.0	99.7	99.9	99.7	98.4	100.0
HDL* Cholesterol	–	26.9	32.9	41.3	45.2	99.6	99.9	99.7	99.7	99.6	98.4	100.0
Glucose	78.6	32.5	99.9	99.8	99.6	99.5	–	99.7	80.4	99.7	98.4	99.9
AST^2^*	99.8	99.9	100.0	99.9	99.6	99.6	100.0	99.7	99.8	99.7	98.4	100.0
ALT^3^*	99.8	99.9	100.0	99.9	99.6	99.6	100.0	99.7	99.8	99.7	98.4	100.0
*γ*-GT^4^*	54.2	26.9	99.9	16.8	45.2	99.6	100.0	99.7	99.9	99.7	98.4	100.0
Uric Acid	–	26.9	99.9	5.0	–	–	–	69.4	54.9	–	98.4	100.0
Creatinin	–	26.9	–	–	45.2	99.6	100.0	99.7	99.8	99.7	98.4	100.0
Anemia												
Hemoglobin	99.9	25.4	99.9	99.9	99.6	99.6	100.0	98.7	99.8	99.7	98.4	100.0
Hematocrit	78.6	25.4	99.9	99.8	99.6	99.6	100.0	–	99.7	99.7	98.4	100.0
Red Blood Cell	21.3	25.4	99.9	99.9	99.4	99.6	100.0	–	99.7	99.7	98.4	100.0
Urinalysis												
Protein	99.5	99.0	98.2	98.3	99.1	98.5	99.9	98.9	99.1	98.3	99.8	99.5
Glucose	99.5	98.9	98.3	98.3	99.1	98.5	99.9	98.9	99.1	97.9	99.8	99.4
Occult Blood	78.5	97.4	86.7	98.3	96.9	96.3	99.0	98.9	98.6	95.2	99.8	99.5

–: not examined

* : high density lipoprotein 2*: aspartate aminotransferase 3*: alanine aminotransferase 4*: *γ*-glutamyl transferase

The ages of the subjects in Katsushika and Suita sub-cohort 1 were just 40 or 50 years old, so the exact comparisons with other populations seem impossible in these two populations. So the descriptions concerning to these populations are not done in the results.﻿﻿

**Table 2.** Mean and standard deviation (S.D.) of height (cm) by sex and age in each population.

Men													
Age (years)		Ninohe	Yokote	Saku	Ishikawa	Katsushika	Kasama	Kashiwazaki	Tosayamada	Arikawa	Miyako	Suita 1	Suita 2
40-49	Mean	163.3	164.6	165.8	162.7	168.7	165.1	164.6	165.7	166.1	163.3	169.8	168.4
	S.D.	5.81	6.07	5.81	5.54	5.54	5.85	5.99	5.44	6.09	6.01	5.69	5.03
50-59	Mean	160.9	161.2	162.6	160.4	165.3	162.3	160.2	163.6	163.0	159.3	166.9	165.3
	S.D.	5.86	5.85	5.47	5.71	5.90	5.83	5.43	6.25	5.48	5.79	5.90	5.36
60-69	Mean	–	–	–	–	–	159.8	158.3	161.4	161.4	157.7	–	163.8
	S.D.	–	–	–	–	–	5.92	5.8	5.52	5.88	5.52	–	5.61

40-59	Mean	161.9	162.7	164.0	161.4	167.0	163.5	161.4	164.3	164.2	160.7	168.3	166.6
	S.D.	5.96	6.19	5.85	5.75	5.98	6.00	5.90	6.05	5.92	6.17	5.98	5.44

Women													
40-49	Mean	151.5	152.7	153.4	149.8	156.1	153.3	153.4	153.5	152.7	151.8	156.8	155.3
	S.D.	5.33	5.46	5.04	4.91	5.35	5.30	4.97	5.36	5.07	5.11	4.83	5.05
50-59	Mean	149.1	150.4	150.5	148.5	153.4	150.8	149.4	151.6	151.9	148.7	154.0	153.0
	S.D.	5.38	5.28	5.08	4.91	5.22	5.09	5.41	5.01	5.11	5.13	5.14	4.79
60-69	Mean	–	–	–	–	–	147.6	146.6	149.3	149.5	147.4	–	151.0
	S.D.	–	–	–	–	–	5.43	5.33	4.96	5.02	5.13	–	5.03

40-59	Mean	150.1	151.3	151.7	149.0	154.5	152.0	150.2	152.4	152.1	149.8	155.3	154.0
	S.D.	5.48	5.46	5.26	4.95	5.43	5.35	5.55	5.24	5.11	5.33	5.18	5.03

**Table 3.** Mean and standard deviation (S.D.) of body weight (kg) by sex and age in each population.

Men													
Age (years)		Ninohe	Yokote	Saku	Ishikawa	Katsushika	Kasama	Kashiwazaki	Tosayamada	Arikawa	Miyako	Suita 1	Suita 2
40-49	Mean	63.3	63.8	64.4	65.9	67.0	64.8	62.5	63.6	65.7	68.8	66.8	66.1
	S.D.	8.98	8.48	8.56	9.66	9.49	9.08	8.06	9.47	9.60	10.32	8.73	8.86
50-59	Mean	60.8	60.8	62.1	62.8	63.9	62.6	58.8	63.3	63.0	64.1	65.5	64.3
	S.D.	8.53	8.25	8.45	9.57	8.53	8.61	7.49	8.82	9.54	9.33	9.01	8.19
60-69	Mean	–	–	–	–	–	58.4	55.3	59.4	59.9	59.6	–	61.0
	S.D.	–	–	–	–	–	8.42	7.59	8.59	9.15	8.78	–	9.02

40-59	Mean	62.0	62.1	63.1	64.0	65.4	63.5	59.8	63.4	64.1	65.7	66.2	65.1
	S.D.	8.82	8.49	8.57	9.71	9.14	8.88	7.78	9.06	9.64	9.93	8.89	8.51

Women													
40-49	Mean	54.6	54.1	54.7	54.9	54.3	54.6	53.5	53.9	54.1	56.5	53.2	53.3
	S.D.	7.74	7.65	7.15	8.27	8.33	8.00	7.87	7.68	7.37	8.76	7.32	7.29
50-59	Mean	53.5	54.1	53.5	55.3	54.1	54.4	52.6	53.9	55.0	55.1	53.4	52.4
	S.D.	7.79	7.86	7.29	8.61	7.72	7.73	6.97	8.03	8.17	8.31	7.16	7.43
60-69	Mean	–	–	–	–	–	52.0	50.6	51.8	52.6	52.9	–	51.5
	S.D.	–	–	–	–	–	8.00	6.81	8.05	8.28	8.01	–	7.96

40-59	Mean	54.0	54.1	54.0	55.1	54.2	54.5	52.7	53.9	54.7	55.6	53.3	52.8
	S.D.	7.79	7.78	7.25	8.47	7.98	7.86	7.15	7.88	7.91	8.50	7.24	7.38

**Table 4.** Mean and standard deviation (S.D.) of body mass index (kg/m^2^) by sex and age in each population.

Men													
Age(years)		Ninohe	Yokote	Saku	Ishikawa	Katsushika	Kasama	Kashiwazaki	Tosayamada	Arikawa	Miyako	Suita 1	Suita 2
40-49	Mean	23.7	23.5	23.4	24.8	23.5	23.8	23.1	23.1	23.8	25.7	23.1	23.3
	S.D.	2.94	2.81	2.68	3.33	3.10	2.91	2.71	2.97	3.02	3.29	2.79	2.76
50-59	Mean	23.5	23.3	23.5	24.4	23.4	23.7	22.9	23.6	23.7	25.2	23.5	23.5
	S.D.	2.78	2.68	2.80	3.15	2.85	2.74	2.69	2.90	2.95	3.04	2.76	2.63
60-69	Mean	–	–	–	–	–	22.8	22.0	22.8	22.9	23.9	–	22.7
	S.D.	–	–	–	–	–	2.82	2.62	2.78	2.90	2.97	–	2.90

40-59	Mean	23.6	23.4	23.4	24.6	23.5	23.7	22.9	23.4	23.7	25.4	23.3	23.4
	S.D.	2.85	2.74	2.75	3.23	2.97	2.81	2.68	2.93	2.97	3.14	2.78	2.69

Women													
40-49	Mean	23.8	23.2	23.3	24.4	22.3	23.2	22.7	22.9	23.2	24.5	21.6	22.1
	S.D.	3.18	3.03	2.91	3.46	3.16	3.11	3.20	3.04	2.95	3.65	2.92	2.81
50-59	Mean	24.1	23.9	23.6	25.1	23.0	24.0	23.5	23.4	23.8	24.9	22.5	22.4
	S.D.	3.19	3.20	2.90	3.53	3.10	3.18	2.67	3.29	3.19	3.30	2.86	2.97
60-69	Mean	–	–	–	–	–	23.9	23.5	23.2	23.5	24.3	–	22.6
	S.D.	–	–	–	–	–	3.32	2.71	3.31	3.33	3.38	–	3.25

40-59	Mean	24.0	23.6	23.5	24.8	22.7	23.6	23.4	23.2	23.6	24.7	22.1	22.3
	S.D.	3.19	3.15	2.91	3.51	3.14	3.16	2.79	3.2	3.12	3.43	2.91	2.89

## RESULTS

### 1. Height, weight and body mass index (BMI) (= body weight (kg) /height^2^ (m)) (Table 2-4)

In both sexes, height was taller in Suita, an urban population, and less in Ishikawa, Ninohe, Miyako and Kashiwazaki. Body weight in men was heavier in Miyako and Suita, and lower in Kashiwazaki, Yokote and Ninohe. In women, it was heavier in Miyako and Ishikawa, and less in Kashiwazaki and Suita. BMI was markedly larger in the two populations in Okinawa in both sexes.

**Table 5.** Mean and standard deviation (S.D.) of systolic blood pressure (mmHg) by sex and age in each population.

Men													
Age (years)		Ninohe	Yokote	Saku	Ishikawa	Katsushika	Kasama	Kashiwazaki	Tosayamada	Arikawa	Miyako	Suita 1	Suita 2
40-49	Mean	132.3	127.8	123.0	128.7	126.6	130.9	133.8	126.6	125.0	129.3	117.2	119.1
	S.D.	15.9	15.1	16.0	15.7	15.8	16.3	21.7	17.6	16.5	16.2	13.7	15.7
50-59	Mean	139.5	134.2	129.2	134.9	132.0	135.7	139.9	131.6	133.1	134.4	124.4	127.6
	S.D.	19.0	18.0	17.0	18.0	18.6	17.6	19.6	18.0	19.5	17.7	17.3	20.9
60-69	Mean	–	–	–	–	–	139.1	138.5	138.0	139.0	135.8	–	133.0
	S.D.	–	–	–	–	–	16.2	18.2	20.8	20.6	17.8	–	20.9

40-59	Mean	136.3	131.4	126.5	132.4	129.4	133.6	138.2	129.8	130.0	132.6	120.9	124.0
	S.D.	18.0	17.1	16.9	17.4	17.5	17.2	20.3	18.0	18.8	17.3	16.0	19.3

Women													
40-49	Mean	127.4	122.4	119.5	122.8	118.7	124.0	124.7	123.3	122.0	121.3	110.4	117.2
	S.D.	16.2	15.1	15.3	15.5	15.5	15.2	16.9	17.4	17.2	17.0	14.0	17.1
50-59	Mean	135.2	131.1	127.0	130.4	128.4	132.5	133.3	130.0	131.4	127.5	119.1	124.9
	S.D.	18.3	17.1	17.0	17.6	19.1	16.5	18.2	20.0	19.1	18.9	18.3	19.7
60-69	Mean	–	–	–	–	–	136.8	140.9	135.9	138.1	132.9	–	132.9
	S.D.	–	–	–	–	–	16.4	18.8	20.6	19.8	18.6	–	20.9

40-59	Mean	131.9	127.7	123.8	127.3	124.3	128.4	131.6	127.3	128.0	125.3	115.0	121.6
	S.D.	17.8	16.9	16.7	17.2	18.3	16.4	18.2	19.3	19.0	18.5	17.0	19.6

**Table 6.** Mean and standard deviation (S.D.) of diastolic blood pressure (mmHg) by sex and age in each population.

Men													
Age (years)		Ninohe	Yokote	Saku	Ishikawa	Katsushika	Kasama	Kashiwazaki	Tosayamada	Arikawa	Miyako	Suita 1	Suita 2
40-49	Mean	78.7	77.7	79.2	82.2	77.5	80.3	81.1	78.1	79.8	81.6	72.3	78.8
	S.D.	10.7	10.5	11.1	10.2	11.1	10.7	12.2	11.1	12.0	11.7	9.9	12.4
50-59	Mean	81.2	81.1	81.5	82.8	81.3	82.3	83.6	80.8	81.3	81.2	77.3	81.1
	S.D.	11.0	10.4	11.0	10.0	11.9	11.2	11.9	11.9	12.2	10.6	11.6	12.6
60-69	Mean	–	–	–	–	–	80.4	81.4	80.1	81.5	78.1	–	80.5
	S.D.	–	–	–	–	–	10.3	11.1	11.2	11.2	10.0	–	11.7

40-59	Mean	80.0	79.6	80.5	82.6	79.5	81.5	82.9	79.8	80.7	81.4	74.8	80.1
	S.D.	11.0	10.6	11.1	10.0	11.7	11.1	12.0	11.7	12.1	11.0	11.1	12.6

Women													
40-49	Mean	74.0	73.8	75.3	76.7	71.0	75.1	76.9	75.2	75.3	74.6	66.7	74.8
	S.D.	9.9	9.6	10.1	10.3	9.9	10.5	11.0	11.1	11.4	11.0	9.4	10.8
50-59	Mean	76.6	78.0	79.3	79.7	76.8	78.7	79.3	77.2	79.4	76.3	71.7	77.6
	S.D.	10.3	10.3	10.5	10.0	11.7	10.5	10.2	11.0	11.3	10.8	11.7	11.6
60-69	Mean	–	–	–	–	–	78.4	80.3	77.4	79.5	76.8	–	78.4
	S.D.	–	–	–	–	–	10.2	11.2	11.4	10.9	10.2	–	10.9

40-59	Mean	75.5	76.3	77.6	78.4	74.4	77.0	78.8	76.4	77.9	75.7	69.4	76.4
	S.D.	10.2	10.2	10.5	10.2	11.3	10.6	10.4	11.0	11.5	10.9	11.0	11.3

### 2. Blood pressure (Table 5,6)

Systolic blood pressure was higher in Kashiwazaki and Ninohe and lower in Suita in both sexes. For ages from 40 to 59, systolic blood pressure showed the greatest difference between the highest in Kashiwazaki and the lowest in Suita (14.2 mmHg in men and 10.0 mmHg in women).

Diastolic blood pressure in men was higher in Kashiwazaki (82.9mmHg) and in Ishikawa (82.6mmHg). For other populations, the values were between 79.5 and 81.4 mmHg. For women, the range was smaller and between 75.5 and 78.8 mmHg.﻿﻿

**Table 7.** Mean and standard deviation (S.D.) of serum total cholesterol (mg/dl) by sex and age in each population.

Men													
Age (years)		Ninohe	Yokote	Saku	Ishikawa	Katsushika	Kasama	Kashiwazaki	Tosayamada	Arikawa	Miyako	Suita 1	Suita 2
40-49	Mean	191.8	193.5	191.7	211.5	203.0	197.5	208.3	201.5	190.3	197.7	200.3	196.2
	S.D.	35.0	33.8	33.7	38.1	36.1	36.2	37.2	39.3	37.3	36.3	33.5	30.5
50-59	Mean	188.9	188.8	190.7	207.8	204.9	193.1	194.9	199.6	189.7	197.0	207.6	197.4
	S.D.	35.4	31.6	32.7	37.1	37.4	34.3	38.7	35.2	34.3	31.3	33.6	30.0
60-69	Mean	–	–	–	–	–	188.9	193.3	194.0	185.5	197.6	–	195.9
	S.D.	–	–	–	–	–	32.9	31.7	33.6	33.6	34.2	–	33.3

40-59	Mean	190.2	190.9	191.1	209.3	204.0	195.0	198.6	200.3	189.9	197.2	204.0	196.9
	S.D.	35.2	32.7	33.1	37.5	36.8	35.2	38.6	36.7	35.5	33.1	33.7	30.2

Women													
40-49	Mean	188.7	192.1	189.3	201.8	189.1	199.0	198.5	199.7	189.4	190.7	189.5	195.5
	S.D.	34.2	33.4	33.3	35.3	31.7	32.9	33.1	34.7	31.0	31.9	29.1	31.0
50-59	Mean	205.3	210.4	210.4	221.9	210.7	216.5	220.0	215.8	204.0	211.2	216.2	216.0
	S.D.	34.2	34.5	36.2	37.5	35.3	33.6	36.2	34.7	34.4	34.6	34.5	32.8
60-69	Mean	–	–	–	–	–	214.7	221.3	213.4	204.7	212.5	–	216.9
	S.D.	–	–	–	–	–	33.8	31.9	33.6	34.2	32.8	–	31.4

40-59	Mean	198.2	203.2	201.4	213.6	201.7	208.1	215.7	209.1	198.9	204.0	203.8	207.1
	S.D.	35.2	35.2	36.5	37.9	35.5	34.4	36.6	35.6	34.0	35.1	34.7	33.6

**Table 8.** Mean and standard deviation (S.D.) of serum triglyceride (mg/dl) by sex and age in each population.

Men													
Age (years)		Ninohe	Yokote	Saku	Ishikawa	Katsushika	Kasama	Kashiwazaki	Tosayamada	Arikawa	Miyako	Suita 1	Suita 2
40-49	Mean	152.7	130.1	156.2	173.2	153.4	168.7	142.2	151.9	118.1	174.4	133.4	141.4
	S.D.	97.2	88.8	116.0	131.5	110.0	133.6	77.8	126.4	104.9	151.6	89.5	98.3
50-59	Mean	129.0	111.8	144.1	188.3	152.5	160.8	147.1	147.8	106.5	141.5	139.1	137.6
	S.D.	76.3	80.0	107.7	152.1	108.3	104.7	172.3	92.7	76.0	138.4	109.4	106.1
60-69	Mean	–	–	–	–	–	138.2	107.6	129.5	97.2	117.7	–	129.0
	S.D.	–	–	–	–	–	85.8	65.4	75.5	61.1	92.0	–	76.4

40-59	Mean	141.9	121.3	149.4	183.0	153.0	164.2	145.7	149.3	111.0	152.8	136.3	139.2
	S.D.	88.9	85.1	111.5	144.8	109.1	118.1	151.3	106.1	88.5	143.9	100.1	102.9

Women													
40-49	Mean	97.5	79.7	98.6	133.3	82.1	114.6	93.0	98.5	80.1	100.4	73.8	85.1
	S.D.	47.1	48.5	63.8	100.0	45.1	77.9	43.4	58.8	46.9	108.6	36.4	50.8
50-59	Mean	121.7	92.8	120.3	157.2	104.6	146.7	124.9	120.9	97.3	116.5	87.2	102.2
	S.D.	67.1	44.1	74.4	93.8	60.4	81.2	70.5	66.0	54.7	75.6	45.2	67.1
60-69	Mean	–	–	–	–	–	147.1	133.8	128.4	101.4	113.9	–	115.5
	S.D.	–	–	–	–	–	80.6	86.1	73.6	56.3	64.8	–	66.3

40-59	Mean	108.5	87.2	111.0	148.4	95.2	131.4	118.6	111.6	91.2	110.8	81.0	94.8
	S.D.	58.3	46.4	70.9	96.6	55.6	81.2	67.1	64.0	52.7	88.9	41.9	61.1

**Table 9.** Mean and standard deviation (S.D.) of serum high density lipoprotein (HDL) cholesterol (mg/dl) by sex and age in each population.

Men													
Age (years)		Ninohe	Yokote	Saku	Ishikawa	Katsushika	Kasama	Kashiwazaki	Tosayamada	Arikawa	Miyako	Suita 1	Suita 2
40-49	Mean		52.8	53.2	51.7	51.5	53.6	59.0	56.0	53.2	54.4	49.8	52.4
	S.D.	–	15.2	13.4	12.7	13.3	14.7	8.9	15.5	15.6	15.1	12.0	13.3
50-59	Mean	–	54.1	55.9	53.0	51.7	52.5	59.7	53.2	55.4	55.7	49.9	52.4
	S.D.	–	15.2	15.0	14.0	13.6	14.8	16.6	15.1	16.8	14.5	13.5	12.9
60-69	Mean	–	–	–	–	–	54.0	61.0	53.3	54.1	57.5	–	53.7
	S.D.	–	–	–	–	–	16.0	16.6	13.7	16.3	15.4	–	14.3

40-59	Mean	–	53.4	54.6	52.5	51.6	53.0	59.5	54.2	54.5	55.3	49.8	52.4
	S.D.	–	15.2	14.3	13.5	13.4	14.8	14.8	15.3	16.4	14.7	12.8	13.0

Women													
40-49	Mean	–	56.6	58.9	54.8	62.1	61.7	58.6	61.5	58.4	57.0	58.9	61.3
	S.D.	–	12.7	12.1	13.0	14.9	15.0	12.0	13.7	14.3	12.3	12.3	13.2
50-59	Mean	–	55.2	60.3	56.3	61.2	58.6	58.6	58.2	56.0	57.7	60.0	61.3
	S.D.	–	12.2	13.1	13.5	15.6	15.3	16.2	13.9	14.7	12.9	13.8	13.6
60-69	Mean	–	–	–	–	–	57.6	58.5	55.6	54.8	57.7	–	59.9
	S.D.	–	–	–	–	–	14.7	14.6	13.5	14.0	12.9	–	13.3

40-59	Mean	–	55.8	59.7	55.7	61.6	60.1	58.6	59.6	56.8	57.4	59.5	61.3
	S.D.	–	12.5	12.7	13.3	15.3	15.2	15.4	13.9	14.6	12.7	13.2	13.4

### 3. Serum total cholesterol, triglycerides and HDL cholesterol levels (Table 7-9)

In men, serum total cholesterol was higher in Ishikawa and Suita, and lower in Yokote, Arikawa, Ninohe and Saku. Difference between the mean values in the highest population, Ishikawa, and the lowest, Arikawa was 19.4 mg/dl in the 40 to 59 year-old range. In women, higher values were found in Kashiwazaki and Ishikawa, and lower in Ninohe and Arikawa. The difference in the highest population, Kashiwazaki, and the lowest, Ninohe, was 17.5 mg/dl.

Serum triglyceride level in men was highest in Ishikawa and lowest in Arikawa. For the levels in men 40 to 59 years old, mean value was 183.0mg/dl in Ishikawa and 111.0mg/dl in Arikawa, and for all the other populations the values were between 121.3 and 164.2mg/dl. In women, the level was higher in Ishikawa and Kasama, and lower in Suita, Yokote and Arikawa. For the levels in 40 to 59 years old, mean value was 148.4mg/dl in Ishikawa, 131.4mg/dl in Kasama and 87.2mg/dl in Yokote, and in all the other populations the values were between 94.8 and 118.6mg/dl.

Serum HDL-cholesterol was measured in all the populations except Ninohe. In men, it was highest in Kashiwazaki. For the levels in 40 to 59 years old, mean value was 59.5mg/dl in Kashiwazaki, and for all the other populations the values were between 52.4 and 55.3mg/dl. In women, it was higher in Kasama and Suita, and lower in Ishikawa. For the levels in women 40 to 59 years old, mean value was 60.1mg/dl in Kasama and 61.3mg/dl in Suita, and in all the other populations, the values were between 55.7 and 59.7mg/dl.

**Table 10.** Mean and standard deviation (S.D.) of blood hemoglobin concentration (g/dl) by sex and age in each population.

Men													
Age (years)		Ninohe	Yokote	Saku	Ishikawa	Katsushika	Kasama	Kashiwazaki	Tosayamada	Arikawa	Miyako	Suita 1	Suita 2
40-49	Mean	15.3	15.3	15.7	15.9	15.7	15.2	14.9	15.0	15.8	15.7	15.2	15.1
	S.D.	1.17	1.16	1.05	0.95	1.16	1.24	1.13	1.03	1.24	1.18	1.00	1.00
50-59	Mean	14.9	14.9	15.4	15.6	15.5	14.9	14.5	14.8	15.3	15.4	15.2	14.9
	S.D.	1.24	1.08	1.21	1.26	1.19	1.10	1.41	1.11	1.41	1.05	1.00	1.21
60-69	Mean	–	–	–	–	–	14.4	13.8	14.4	15.1	15.0	–	14.6
	S.D.	–	–	–	–	–	1.20	1.42	1.23	1.45	1.21	–	1.28

40-59	Mean	15.1	15.1	15.5	15.7	15.6	15.0	14.6	14.9	15.5	15.5	15.2	15.0
	S.D.	1.22	1.13	1.15	1.16	1.18	1.17	1.35	1.08	1.37	1.10	1.00	1.13

Women													
40-49	Mean	12.9	12.6	13.1	13.1	13.0	12.7	12.5	12.7	13.2	13.0	12.5	12.4
	S.D.	1.31	1.60	1.49	1.51	1.44	1.45	1.52	1.31	1.44	1.38	1.41	1.50
50-59	Mean	13.2	13.2	13.7	13.6	13.3	13.2	12.9	13.0	13.5	13.5	12.8	13.0
	S.D.	1.03	0.99	1.01	1.00	1.43	0.99	1.04	0.89	1.16	1.06	1.46	1.03
60-69	Mean	–	–	–	–	–	13.0	12.7	12.9	13.4	13.4	–	12.9
	S.D.	–	–	–	–	–	1.02	1.01	0.95	1.15	1.03	–	1.11

40-59	Mean	13.1	12.9	13.5	13.4	13.2	12.9	12.8	12.9	13.4	13.3	12.6	12.7
	S.D.	1.17	1.32	1.27	1.26	1.44	1.26	1.16	1.10	1.27	1.21	1.44	1.28

### 4. Blood hemoglobin concentration (Table 10)

Blood hemoglobin concentration in men was highest in Ishikawa. For the levels in men 40 to 59 years old, mean value was 15.7g/dl in Ishikawa, and for all the other populations the values were between 14.6 and 15.5g/dl. In women, the difference was smaller than in men, and the mean value in each population was between 12.7 to 13.5g/dl.

## DISCUSSION

Standardization of data collection was not done systematically in this survey for blood pressure measurements and serum lipid measurements, but all the laboratories had attended individually the standardization system operated by Japanese medical association. Center for Disease Control (CDC) of USA standardization methodology for the measurement of serum total cholesterol level was applied for the Cohort II populations by Iida et al^1^^)^. Standardization of the data collection methodology and the measurement of serum lipid level will be made for all the populations in the intermediate 5th year examination of this cohort study.

Comparisons were made for the data from the Japanese national surveys and American National Health and Nutrition Examination Surveys (NHANES) (Table 11)^2^^)^. Systolic blood pressure was generally less than the value given in the Japanese national survey in 1990, and was similar to that of US whites in NHANES II data in men and women^3^^)^. Diastolic blood pressure was less than the values in the Japanese national surveys and NHANES II. The examination years in the present survey are between 1990 and 1993, so the data may be similar to the data of the Japanese national survey in 1990. However, the present result showed a lower value. Sampled population might have caused the difference, but the values this time are lower than any divided districts in the national survey report. The sample sizes were bigger in each area in the present survey, in contrast to the smaller size in each district in the national survey. One possible reason might be that the populations in this survey happened to be chosen lower blood pressure areas. Another possible reason might be the bias due to the attendance rate of the response rate in this survey being lower than in the national survey.

**Table 11.** Mean and standard deviation (S.D.) of blood pressure (mmHg) by sex and age in Japanese and American national surveys.

	Systolic blood pressure (mmHg)		Men	Diastolic blood pressure (mmHg)		
Age (years)						
National surveys	40-49	50-59	60-69	40-49	50-59	60-69
	Mean (S.D.)	Mean (S.D.)	Mean (S.D.)	Mean (S.D.)	Mean (S.D.)	Mean (S.D.)

Japanese*	132.4 (16.9)	139.4 (19.8)	145.4 (20.9)	83.5 (11.4)	85.8 (12.1)	85.7 (12.0)
US whites**	127.9 (16.7)	135.6 (20.9)	140.7 (22.3)	85.1 (12.1)	87.4 (12.8)	85.2 (12.2)

Women						
Japanese	127.8 (17.0)	136.7 (19.5)	143.5 (19.1)	79.0 (11.2)	82.9 (11.7)	82.7 (11.4)
US whites	123.1 (19.0)	134.4 (21.0)	141.3 (23.8)	80.7 (12.9)	84.0 (12.8)	83.7 (12.4)

* : Japanese National Survey of Circulatory Disorders, 1990

** : The second National Health and Nutrition Examination Survey of the US (NHANES II), 1976-1980

Serum total cholesterol level was just less than that of the Japanese national data^1^^)^ in 1990, and NHANES III data^4^^)^ in 1990. The reason may be similar to the one just mentioned concerning to blood pressure level. However, strictly, this has to be concluded after the standardization of the laboratory data will be finished in the intermediate year examination.
